# Circulating Tumor HPV‐DNA in the Management of HPV‐Positive Oropharyngeal Carcinoma: A Systematic Review

**DOI:** 10.1002/hed.28057

**Published:** 2025-01-23

**Authors:** Allen M. Chen, Tjoson Tjoa, William B. Armstrong

**Affiliations:** ^1^ Department of Radiation Oncology University of California, Irvine, Chao Family Comprehensive Cancer Center Orange California USA; ^2^ Department of Otolaryngology University of California, Irvine, Chao Family Comprehensive Cancer Center Orange California USA

**Keywords:** biomarker, cell‐free, circulating DNA, HPV, liquid biopsy

## Abstract

**Purpose:**

Blood‐borne, cell‐free DNA has been proposed as a means of individualizing the management of human papillomavirus (HPV)‐positive oropharyngeal carcinoma.

**Methods and Materials:**

This study was designed based on the Preferred Reporting Items for Systematic Review and Meta‐Analysis Protocols (PRISMA‐P) statement. A comprehensive literature search of peer‐reviewed publications from January 2013 to January 2024 was undertaken to identify prospective studies pertaining to the use of circulating HPV‐DNA for oropharyngeal carcinoma.

**Results:**

A total of 11 prospective studies were identified and differed in their clinical design, methods, and endpoints. Five included patients treated by chemoradiation; 3 by surgery; 2 by both; and 1 not specified. The timing and frequency of HPV‐DNA draws was highly variable. The sample size ranged from 16 to 262 (mean, 99 patients).

**Conclusions:**

While interest is growing with integrating circulating HPV‐DNA into clinical practice, the supporting evidence is limited by the heterogeneity of the evidence.

## 1 Introduction

The worldwide incidence of human papillomavirus (HPV)‐associated oropharyngeal squamous cell carcinoma has increased dramatically in recent years reaching epidemic‐like proportions in developed countries [1]. Although newly diagnosed patients with HPV‐positive oropharyngeal cancer have an improved prognosis compared to those with HPV‐negative disease, it is clear that a certain subset of patients have biologically more aggressive tumors placing them at higher risk for recurrence [2]. Given emerging data which have questioned traditional risk factors for disease relapse for patients with HPV‐positive oropharyngeal cancer, the need to identify novel markers of prognostic significance which can be integrated into clinical practice is imperative [3]. This is particularly the case given the increased interest in the use of de‐escalation which aims to reduce the intensity of treatment. The ability to reliably predict which patients might be most appropriate for such strategies both at baseline and mid‐treatment could rigorously refine selection criteria and better stratify therapeutic options for patients with HPV‐positive oropharyngeal cancer.

In this regard, the use of blood‐borne, cell‐free DNA to quantify disease burden and to longitudinally monitor response has been proposed as a potentially dynamic means of treatment individualization. By quantitatively assessing HPV‐DNA levels in serial fashion—at diagnosis, during treatment, and at various points in the post‐treatment setting—this liquid biopsy assay has the potential to acquire tumor‐specific data and to analyze kinetic information related to HPV‐DNA levels which could improve prognostication and also identify patients at risk for disease recurrence. While this technology has exploded in popularity in recent years, as if potentially offers a more precise, biologically driven approach to treatment and surveillance, the evidence supporting its adoption remains preliminary. Indeed, numerous unanswered questions persist, and considerable uncertainty exists on how to optimally integrate this technology into clinical practice. The purpose of this review is to thus outline the available evidence to date focusing on prospectively acquired data evaluating the utility of circulating HPV‐DNA for patients with HPV‐positive oropharyngeal cancer.

## 2 Methods and Materials

This study was designed based on the Preferred Reporting Items for Systematic Review and Meta‐Analysis Protocols (PRISMA‐P) statement. A comprehensive literature search of peer‐reviewed publications was undertaken to identify original peer‐reviewed works pertaining to the use of liquid biopsy technologies and/or circulating HPV‐DNA assays for local‐regionally confined squamous cell carcinoma of the oropharynx. Given the goal of critically evaluating high‐level evidence which could enable the adoption of such technologies into clinical practice, the focus of this work was on specifically identifying prospective studies. The initial screen was conducted on January 14, 2024, and repeated again on February 14, 2024.

Reference lists from included articles were cross‐checked to identify additional articles. Review articles and papers presented as conference proceedings were excluded. Articles published from January 2014 to January 2024 with full text available on PubMed and restricted to the English language and human subjects were included. The full bibliographies of identified articles were reviewed and irrelevant studies including those focused exclusively on the diagnostic work up (e.g., the correlation of circulating HPV‐DNA with clinical and/or radiological findings at baseline) and/or utilizing circulating HPV‐DNA as screening methods in subjects without a cancer diagnosis were selectively removed. Studies that were designed as retrospective analysis of data that had been acquired during the course of patient care were also excluded. Where individual patients were included in multiple published series, the most complete or recent article was cited. An interpretive synthesis of the available publications was then presented focused on presenting the prospective evidence evaluating the role of circulating HPV‐DNA in the clinical management of oropharyngeal cancer.

## 3 Results

### 3.1 Search Results

**FIGURE 1:**
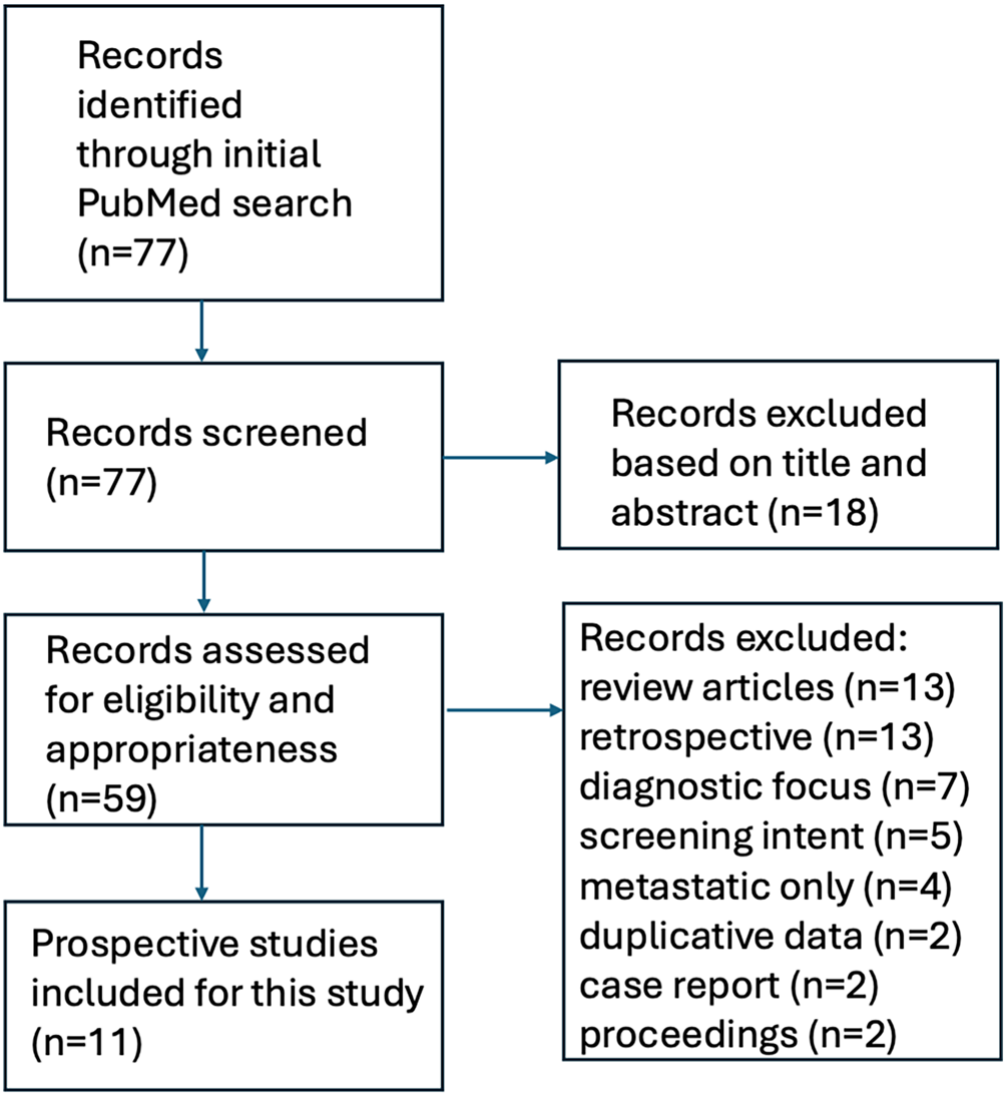
Schematic illustration of the flowchart outlining the results of the search strategy. [Color figure can be viewed at wileyonlinelibrary.com]

The initial search yielded 77 articles. After screening of these articles on title and abstract, a total of 59 studies proceeded to full‐text screening. Another 48 articles were excluded because they were review articles (*N* = 13), were retrospective in nature (*N* = 13), more focused on diagnostic correlation with clinical and/or radiological findings at baseline (*N* = 7), were designed as screening studies for subjects without a known cancer diagnosis (*N* = 5), was designed to study patients with distant metastasis (*N* = 4), used duplicative data (*N* = 2), and was designed as a case report (*N* = 2) or conference proceedings (*N* = 2). A total of 11 peer‐reviewed prospective studies thus were included and formed the basis for this systematic review. A schematic illustration of the flowchart outlining the results of the search strategy is shown in Figure 1.

### 3.2 Identified Themes

The 11 prospective studies we identified differed significantly in their clinical design, methods, and endpoints. Five included patients with HPV‐positive oropharyngeal cancer treated by chemoradiation; 3 were surgical series; 2 included a mixed population of both surgically and non‐surgically treated patients; and 1 did not specify how treatment was delivered. The sample size ranged from 16 to 262 (mean, 99 patients). The timing and frequency of HPV‐DNA draws was highly variable. Table 1 outlines the details of the prospective studies and summarizes the key findings from the 11 prospective trials.

**TABLE 1:** Prospective studies evaluating the utility of circulating HPV‐DNA in HPV‐positive oropharyngeal cancer.

Study	*N*	Primary treatment	Collection schedule	Endpoints	Primary finding
Dahlstrom et al.	262	NS	Baseline, 6 weeks then every 3 months for 3 years	PFS	Undetectable HPV‐DNA at baseline associated with improved PFS
Jakobsen et al.	72	Mixed	Baseline, 2 weeks, then 6, 9, 12, 18, 24, 30 months	Recurrence	HPV‐DNA had high sensitivity for post‐treatment follow‐up surveillance
Haring et al.	16	Mixed	Baseline, 3 points during treatment	Radiographic response	HPV‐DNA declines observed earlier than radiographic response and were predictive
Adrian et al.	136	CRT	Baseline and post‐treatment	PFS, OS, LRC	Serial declines in HPV‐DNA associated with improved PFS and OS
Cao et al.	34	CRT	Baseline and weeks 2, 4, 7 during CRT and post‐treatment	FFP	Low baseline HPV‐DNA and increase at week 2 associated with improved FFP
O'Boyle et al.	33	Surgery	Baseline and POD 1, 7, 30	Post‐operative disease status	POD 1 HPV‐DNA levels associated with risk of residual disease
Souza et al.	196	Surgery	Baseline and various post‐treatment points	NPV, PPV or surveillance	High NPV after surgery may identify omission of adjuvant radiation
Routman et al.	32	Surgery	Baseline and POD NS	Recurrence‐free survival	Detectable HPV‐DNA after surgery is associated with decreased survival
Chera et al.	103	CRT	Baseline, weekly during CRT, various post‐treatment points	Clearance profile	Favorable clearance profile may predict for disease control after completion of CRT*
Chera et al.	115	CRT	Baseline, weekly during CRT, various post‐treatment points	NPV, PPV of surveillance	High NPV and PPV shown for predicting disease relapse in post‐treatment setting
Mazurek et al.	91	CRT	Baseline	OS, LRC, DMFS	High pre‐treatment HPV‐DNA levels predict for distant metastasis

Abbreviations: CRT, chemoradiation; DMFS, distant metastasis‐free survival; FFP, freedom from progression; LRC, local‐regional control; NPV, negative predictive value; NS, not specified; OS, overall survival; PFS, progression‐free survival; POD, post‐operative day; PPV, positive predictive value.

* Favorable clearance profile as having high baseline copy number (> 200 copies/mL) and > 95% clearance of ctHPV16DNA by day 28 of CR.

## 4 Discussion

Despite robust interest in the use of liquid biopsy technology in the management of cancer, there is a relatively small number of prospective studies that have evaluated the utility and performance of circulating HPV‐DNA for patients with HPV‐positive oropharyngeal cancer. While such platforms have ushered in a new era of precision care by allowing for the molecular characterization and quantification of HPV‐positive oropharyngeal cancer through minimally invasive means, the limited amount of evidence supporting its usage for clinical decision‐making was quite striking. Although studies touting the benefit of circulating HPV‐DNA to inform clinical decision‐making at varying points of the care continuum including at diagnosis, mid‐treatment, and during surveillance is increasingly heralded, the data is still preliminary at present and continues to evolve.

The rationale behind the impetus to integrate the measurement of circulating HPV‐DNA into practice paradigms is compelling—to add prognostic information, to aid in the stratification of therapy, and to longitudinally monitor patients during and after completion of treatment. At diagnosis, the currently utilized risk stratification models for HPV‐positive oropharyngeal cancer are poorly defined with the only 2 established risk factors being tumor stage via the American Joint Committee on Cancer (AJCC) system and smoking history [4]. For surgically treated patients, the relevance of such traditionally important pathological features such as extracapsular lymph node extension, positive margins, and lympho‐vascular space invasion are uncertain and continue to be debated in the setting of HPV‐positive oropharyngeal cancer [5]. Thus, the addition of HPV‐DNA titers to traditional prognostic models could potentially add a degree of accuracy based on the inherent biological characteristics of the tumor. Similarly, data related to circulating HPV‐DNA kinetics may offer insight into the responsiveness of this cancer to treatment and may even be useful for predicting relapse during or after treatment.

The bulk of the prospective evidence to data has focused on integrating HPV‐DNA data acquired at baseline into prognostic models for oropharyngeal cancer. Dahlstrom et al. showed that patients with higher levels of HPV‐DNA at diagnosis were associated with increased AJCC stages of disease [6]. Although patients with detectable pre‐treatment levels of circulating HPV‐DNA had worse progression‐free survival than patients without detectable circulating HPV‐DNA, this difference did not reach statistical significance. The investigators also suggested that HPV‐DNA levels post‐treatment could be of utility in predicting recurrence. Among the 11 patients who developed distant metastasis, 4 (36%) were shown to have detectable levels of circulating HPV‐DNA following treatment, thereby suggesting that the appearance of these biomarkers could serve as a precursor for recurrence. Notably, among the 17 patients who developed local‐regional recurrence, only 1 (6%) had detectable HPV‐DNA which raised concerns regarding the sensitivity of the assay. In another similar study, Mazurek et al. showed that pre‐treatment levels of circulating HPV‐DNA correlated with the risk of distant metastasis for HPV‐positive oropharyngeal cancer patients after non‐surgical treatment [7]. More recently, Jakobsen et al. conducted a prospective cohort study of 72 patients with HPV‐positive oropharyngeal cancer [8]. Consistent with others, the investigators showed that HPV‐DNA levels at baseline were associated with AJCC stage. Notably, among the 13 patients with persistently detectable HPV‐DNA after curative treatment, a total of 8 patients developed disease relapse. However, the fact that 5 patients in this group never developed any type of recurrence suggesting that the specificity of this assay might be problematic, or alternatively, longer follow up may have been necessary given the slower HPV‐DNA kinetics related to this subset.

The potential utility of mid‐ and post‐treatment levels of HPV‐DNA to serve as a reliable biomarker to predict for response and recurrence for HPV‐positive oropharyngeal cancer is provocative. While investigators have shown that the dynamic monitoring of HPV‐DNA can be feasible through serial blood draws, how to make practical sense of this kinetic data largely remains to be determined. Nonetheless, several studies have been published suggesting applications in which this technology can be integrated into clinical practice [9, 10]. Although the evidence generally shows that patients who reach a nadir in their HPV‐DNA levels more rapidly after initiation of treatment and/or have persistently low (or undetectable) levels of HPV‐DNA thereafter might have an improved prognosis, the ways in which clearance and kinetic response were defined have been highly variable. For instance, in a published a subset analysis of the Swedish ARTSCAN III trial in which patients were randomized to radiation with cisplatin or radiation with cetuximab, the investigators assessed the prognostic significance of changes in HPV‐DNA levels by using an area‐under‐the‐curve (AUC) logarithmic model [11]. Notably, in a multi‐variable analysis considering established risk factors for recurrence, HPV‐DNA clearance via the AUC model was identified as an independent predictor of progression‐free survival. Conversely, Cao et al. showed that an early increase in circulating HPV‐DNA from baseline to week 2 was associated with superior freedom from progression among 34 patients with HPV‐positive oropharyngeal cancer treated by chemoradiation [12]. Given the correlation that was established between HPV‐DNA and apparent diffusion coefficient (a measurement of tumor liquification in response to treatment), the investigators suggested that an early rise in circulating HPV‐DNA was indicative of treatment response.

In the most mature series to date, Chera et al. enrolled 115 patients with HPV‐positive oropharyngeal cancer on a prospective trial investigating the utility of circulating HPV‐DNA in the post‐treatment setting after chemoradiation [13]. With a median follow‐up time of 23 months, a total of 15 patients (13%) developed disease recurrence. Notably, among the 87 patients with undetectable HPV‐DNA at all post‐treatment time points, none developed recurrence—resulting in a negative predictive value of 100%. Conversely, 15 of the 28 patients (54%) who had a detectable HPV‐DNA level during the post‐treatment period developed recurrence. While only 16 patients had 2 consecutive positive HPV‐DNA tests, a striking 15 of them developed recurrence—suggesting that this metric might be the most useful for identifying patients at risk for relapse with the highest sensitivity and specificity. Notably, in an earlier study of 103 patients with HPV‐positive oropharyngeal cancer treated by chemoradiation, the investigators had shown the utility of “favorable clearance profile” through quantitative metrics—as defined by high baseline HPV‐DNA copy number (> 200 copies/mL) and > 95% clearance of circulating HPV‐DNA by day 28 of treatment [14]. Nineteen of the 67 evaluable patients displayed a favorable clearance profile, and none had persistent or recurrent regional disease after chemoradiation. In contrast, patients with adverse clinical risk factors (T4 or > 10 pack years) and unfavorable clearance profile had a 35% rate of persistent or recurrent regional disease after chemoradiation.

Several prospective studies have been published demonstrating the ability of circulating HPV‐DNA kinetics to predict for patients at risk for recurrence after surgery for HPV‐positive oropharyngeal cancer [15, 16, 17]. O'Boyle et al. conducted a prospective trial of 33 patients with HPV‐positive oropharyngeal cancer and showed that HPV‐DNA data could be used to supplement existing pathological data to make decisions regarding adjuvant therapy [15]. The investigators showed that patients without pathologic risk factors for recurrence had HPV‐DNA rapidly decrease to less than1 copy/mL by post‐operative day 1. Moreover, patients with risk factors for macroscopic residual disease had markedly elevated HPV‐DNA levels, defined as greater than 350 copies/mL on post‐operative day 1. The intermediate group of patients who failed to meet criteria for either of these 2 groups all possessed pathologic risk factors for microscopic residual disease. Souza et al. similarly showed that HPV‐DNA obtained after surgery alone could be of utility in predicting patients at high risk for recurrence without adjuvant therapy [16]. In their study of 196 patients, the sensitivity and specificity of an initial post‐surgery HPV‐DNA test in predicting for biopsy‐proven disease was 97% and 60%, respectively. Lastly, in a prospective analysis of 32 patients with HPV‐positive oropharyngeal cancer, Routman et al. showed that detectable levels of HPV‐DNA after surgery were associated with age, lympho‐vascular space invasion, and extracapsular lymph nodal extension [17]. Furthermore, the investigators reported that recurrence‐free survival at 18 months was 83% for patients with detectable post‐operative HPV‐DNA compared with 100% for patients with undetectable levels.

While the emergence of liquid biopsy technology has introduced a potentially innovative way of managing patients with cancer, it must be recognized that the evidence supporting its integration into clinical practice remains somewhat preliminary. Indeed, a recent survey of 90 physicians specializing in the management of HPV‐positive oropharyngeal cancer showed that adoption of circulating HPV‐DNA into current surveillance practices is still controversial [18]. While 57% of respondents reported that they would consider accepting the use of such technology in practice, most recommended its integration starting at year 3 post‐treatment presumably due to concerns regarding false‐positive and false‐negative findings in the more immediate setting. Supporting this belief was the fact that even the prospective studies that we identified were limited by somewhat small sample sizes and used single‐arm designs without control subjects. Furthermore, nearly all of the data was single‐institutional with varying selection criteria. Additionally, the timing of circulating HPV‐DNA assessment was inconsistent across studies which contributed to significant heterogeneity in study design. As a result, the research endpoints varied widely, which limited the ability to compare findings across studies. This was further hampered by relatively short follow up. It was thus hardly surprising that the conclusions drawn by individual studies have varied and, in some cases, could be considered hypothesis generating at best.

Although the use of liquid biopsy techniques to test for the presence of HPV‐DNA in the plasma of patients with HPV‐positive oropharynx cancer is attractive due to its non‐invasive nature and rapid turnaround time, it must be recognized that the methods in use are still in need of large‐scale validation. Indeed, given that circulating DNA can be labile with a short half‐life, consideration should be given to possible problems with consistency and reliability [19]. Inter‐assay variability in the reporting of results, particularly with respect to the quantification of titer levels, have historically limited the use of these technologies [20]. While polymerase chain reaction‐based tests have formed the mainstay of DNA detection, the recent advent of ultra‐sensitive assays have the potential to introduce more variability between detection methods [21, 22]. Thus, studies investigating how to best standardize the methods of HPV detection are urgently needed. Given the relatively limited data to date, research to better define thresholds of practical relevance incorporating mathematical models of clearance would be useful to refine the clinical utility of this technology.

Lastly, the use of biomarkers other than circulating HPV‐DNA need to be evaluated in this setting. In prior studies, HPV‐16 E6/E7 antibodies exhibited a strong association with HPV‐DNA and could be leveraged to further enhance detection methods [23]. Further, investigators evaluating HPV‐positive tumor tissue have reported discovery of unique microRNA expression signatures which could be exploited [24]. Biologically driven approaches using exosomes have also been proposed given that exosomes and their associated micro‐vesicles can possibly be selected to improve the sensitivity and specificity of assays testing for HPV‐DNA alone in the setting of HPV‐positive oropharyngeal cancer [25]. Lastly, the potential for saliva as a medium to assess disease response is attractive given the proximity of the oropharyngeal cancer to the easily accessible oral cavity [26].

In conclusion, the potential for the integration of circulating HPV‐DNA assays into the clinical management of patients with HPV‐positive oropharyngeal cancer is increasingly being recognized. However, few prospective studies have fully evaluated the utility of these platforms in a rigorous and methodical fashion. Consequently, numerous questions persist, particularly with respect to the accuracy, reliability and clinical applicability of the technology. While ongoing scientific advances and clinical trials will further refine the role of circulating HPV‐DNA detection methods, the promises and pitfalls of this technology need to be recognized.

## Conflicts of Interest

The authors declare no conflicts of interest.

## Data Availability

Data sharing is not applicable to this article as no new data were created or analyzed in this study.
